# The Structural Basis of *Erwinia* *rhapontici* Isomaltulose Synthase

**DOI:** 10.1371/journal.pone.0074788

**Published:** 2013-09-19

**Authors:** Zheng Xu, Sha Li, Jie Li, Yan Li, Xiaohai Feng, Renxiao Wang, Hong Xu, Jiahai Zhou

**Affiliations:** 1 State Key Laboratory of Materials–Oriented Chemical Engineering, College of Food Science and Light Industry, Nanjing University of Technology, Nanjing, China; 2 State Key Laboratory of Bio-Organic and Natural Products Chemistry, Shanghai Institute of Organic Chemistry, Chinese Academy of Sciences, Shanghai, China; University of Edinburgh, United Kingdom

## Abstract

Sucrose isomerase NX-5 from *Erwinia* *rhapontici* efficiently catalyzes the isomerization of sucrose to isomaltulose (main product) and trehalulose (by-product). To investigate the molecular mechanism controlling sucrose isomer formation, we determined the crystal structures of native NX-5 and its mutant complexes E295Q/sucrose and D241A/glucose at 1.70 Å, 1.70 Å and 2.00 Å, respectively. The overall structure and active site architecture of NX-5 resemble those of other reported sucrose isomerases. Strikingly, the substrate binding mode of NX-5 is also similar to that of trehalulose synthase from *Pseudomonas* *mesoacidophila* MX-45 (MutB). Detailed structural analysis revealed the catalytic RXDRX motif and the adjacent 10-residue loop of NX-5 and isomaltulose synthase PalI from *Klebsiella* sp. LX3 adopt a distinct orientation from those of trehalulose synthases. Mutations of the loop region of NX-5 resulted in significant changes of the product ratio between isomaltulose and trehalulose. The molecular dynamics simulation data supported the product specificity of NX-5 towards isomaltulose and the role of the loop^330-339^ in NX-5 catalysis. This work should prove useful for the engineering of sucrose isomerase for industrial carbohydrate biotransformations.

## Introduction

Isomaltulose (also known as α-D-glucopyranosyl-1,6-D-fructofuranose or palatinose) and trehalulose (α-D-glucopyranosyl-1,1-D-fructofuranose) are two structural isomers of sucrose. Although they possess similar physical and organoleptic properties as sucrose, they can prevent tooth decay and attenuate the glycemic index and insulin levels in the bloodstream [1-5]. Therefore, they have been identified as valuable sucrose substitutes for obese and diabetic individuals [6]. In addition, the reducing properties of isomaltulose make it attractive as an industrial precursor for the production of biosurfactants and biocompatible polymers [7].

**Figure 1:**
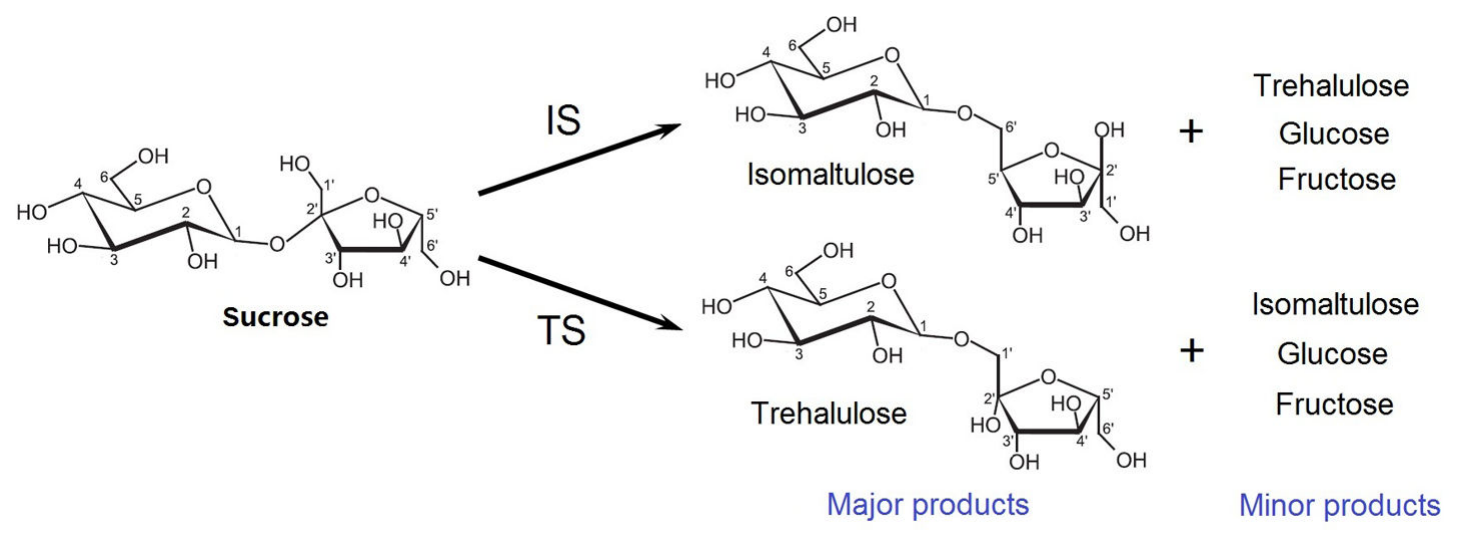
Schematic depictions of the sucrose isomerization process. Sucrose was converted to isomaltulose, trehalulose, D-glucose and D-fructose by isomaltulose synthase (IS) or trehalulose synthase (TS).

In nature, isomaltulose and trehalulose exist in honey and sugar cane in very small quantities. Compared to the low-yielding chemical synthesis process, the biological production of isomaltulose and trehalulose using sucrose isomerases (SIases; EC 5.4.99.11) as catalysts is more practical and economical. SIases, classified as glycoside hydrolase family 13 (GH13) enzymes, catalyze the isomerization of sucrose to produce isomaltulose and trehalulose as primary products and D-glucose and D-fructose as by-products (Figure 1) [8-10]. The overall enzymatic reaction steps consist of sucrose binding, sucrose hydrolysis, glucosyl-enzyme intermediate occurrence, fructose release, and sucrose isomer formation [11-13]. Based on the product preference of sucrose isomers, SIases can be divided into two groups, isomaltulose synthases and trehalulose synthases. The protein sequences of isomaltulose synthases from *Klebsiella* sp. LX3 [14], *Protaminobacter* *rubrum* [15], *Serratia* *plymuthica* [16,17], *Pantoea* *dispersa* UQ68J [18], and *Enterobacter* sp. FMB1 [19] share high homology with those of trehalulose synthases from *Agrobacterium* *radiobacter* [20] and *Pseudomonas* *mesoacidophila* (also known as *Rhizobium* sp. by Goulter et al.) [21-23]. To date, 16 related SIase structures have been deposited and released in the PDB database. There are two native isomaltulose synthase structures including those from *Klebsiella* sp. LX3 (also known as PalI) [12] and *P* *. rubrum* (SmuA) [24], and several complex structures of trehalulose synthases such as, MutB-Tris, MutB-castanospermine, MutB-deoxynojirimycin, MutB D200A-glucose, MutB E254Q-sucrose and MutB-glycerol [13,25]. A structural comparison revealed that isomaltulose synthases and trehalulose synthases possess a very similar overall fold and active site architecture. Differences discovered in the surface charges around the active site entrance are believed to be one of the key factors involved in the sucrose isomerization process [24]. In 2003, Zhang et al. identified a unique catalytic^325^RLDRD^329^ motif in PalI affecting the product specificity of sucrose isomerization [26]. Mutations on any charged residues in this motif dramatically increased the production ratio of trehalulose over isomaltulose [15,26]. Interestingly, the amino acid sequences of this motif are well conserved in isomaltulose synthases but vary between isomaltulose synthases and trehalulose synthases. However, replacement of RLDRD from *Klebsiella pneumoniae* NK33 isomaltulose synthase by^284^RYDRA^288^ from *P* *. mesoacidophila* MX-45 trehalulose synthase reduced its enzymatic activity without changing the product ratio [27]. Although isomaltulose synthases and trehalulose synthases are widely used in industry, their structural differences remains not sufficiently understood.

Previously, we have characterized an isomaltulose synthase from *Erwinia* *rhapontici* NX-5 (named NX-5 in this work) that predominantly produces isomaltulose (~87%) [28,29]. Compared to other isomaltulose synthases, NX-5 shows more acidic tolerance and higher product selectivity on sucrose isomerization. To understand the mechanism of product specificity and the molecular details controlling sucrose isomerization, we determined the crystal structures of native NX-5 and its mutant complexes E295Q/sucrose and D241A/glucose. In combination with mutagenesis and molecular dynamics (MD) simulations, our work provides new insights for elucidating the structural differences of isomaltulose synthases and trehalulose synthases.

## Materials and Methods

### Chemicals, reagents, strains and plasmids

Sucrose, isomaltulose, trehalulose, D-glucose, and D-fructose were obtained from Sigma. Restriction enzymes, T4 DNA ligase, Ex-Taq DNA polymerase, and *Dpn*I were products from Takara (Dalian, China). Vector pET-22b and strain BL21 (DE3) were purchased from Novagen and used for protein expression. All reagents for crystallization optimization were bought from Sigma.

### Gene cloning, protein expression and purification

To facilitate protein crystallization, we modified the recently reported expression plasmid pET-22b-palI by truncating the gene encoding the N-terminal peptide sequence [29]. Briefly, the gene of *Erwinia* *rhapontici* NX-5 was amplified using the primers F: 5’-GGCGTTCCATATGGATTCTCAAGGATTG-3’; R: 5’-CCGCTCGAGCGGATTAAGTTTATAAAT-3’ (*Nde*I and *Xho*I restriction sites are underlined) and pET-22b-palI as the template. The resulting PCR products were digested with *Nde*I/*Xho*I and ligated into pET-22b. The mutations were introduced by PCR using the primers listed in Table S1 in File S1 and followed the QuickChange (Stratagene) protocol. The sequence of each clone was confirmed by DNA sequencing.

The wild-type and mutant NX-5 proteins were expressed in *E. coli* BL21 (DE3) strain as described previously [29] at 297 K by the addition of 0.5 mM lactose in refined culture medium (pH 7.0) consisting of sucrose 10 g/L, yeast extract 20 g/L, NaCl 9 g/L, KH_2_PO_4_ 3 g/L, and MgSO_4_ 0.5 g/L. The cells were harvested by centrifugation after growth for 11 h. Cell pellets were lysed by sonication in buffer A (10 mM HEPES, pH 8.0, 500 mM NaCl, 5 mM β-mercaptoethanol, and 10% glycerol). After centrifugation for 15 min at 10,000 g, the supernatant was loaded onto a nickel-affinity column (GE Healthcare) and washed with buffer containing 10 mM HEPES, pH 8.0, 500 mM NaCl, 5 mM β-mercaptoethanol, 10% glycerol, and 50 mM imidazole. The recombinant protein was eluted from the affinity resin with 500 mM imidazole in buffer A. The NX-5 with a C-terminal His tag was further purified using a Superdex 200 16/60 column (GE Healthcare). The pooled proteins were concentrated with a Centricon 10 (Millipore) and stored in 10 mM HEPES, pH 7.0, 150 mM NaCl, 1 mM DTT, and 10% glycerol. The protein concentration was determined by the Bradford method using bovine serum albumin as the standard [30].

### Sucrose isomerase activity assay

The sucrose isomerase activities of the NX-5 proteins were measured using a high performance liquid chromatography method [29]. In brief, 0.5 mg of purified enzyme solution was mixed with 20 g/L of sucrose in a 1 mL reaction volume containing 50 mM PBS, pH 6.0. Reactions were conducted at 30°C for 3 h. The product samples were analyzed by HPLC (Agilent 1200, USA) equipped with a refractive index detector (Shodex RI100, Japan). D-Glucose, D-fructose, sucrose, trehalulose, and isomaltulose at a concentration of 10 g/L each were used as standards.

### Protein crystallization, data collection, and structure determination

Crystals were grown at 293 K by the sitting-drop vapor diffusion method by mixing 1 µL of NX-5 protein (10 mg/mL) with an equal volume of reservoir solution and equilibrating against 500 µL of reservoir solution in a sealed 24-well plate. Initial screening trials were set up using the Structure Screen I & II HT-96 kits from Molecular Dimensions LTD. Crystals of NX-5 were grown in the presence of 0.1 M trisodium citrate, pH 5.6, 0.2 M ammonium acetate, and 30% PEG 4000. The E295Q/sucrose complex crystals were grown in the presence of 20 mM sucrose, 0.1 M bicine, pH 9.0, and 30% PEG 6000. The D241A/glucose complex was co-crystallized in the presence of 20 mM sucrose, 0.1 M sodium cacodylate, pH 6.5, and 29% PEG 8000. Large and single crystals were usually obtained after 3 to 5 days.

Prior to data collection, crystals were soaked in a cryoprotectant solution containing 80% reservoir solution and 20% glycerol for a few seconds and then flash-frozen in liquid nitrogen. Diffraction data were collected on an ADSC Q315r detector at 100 K at beam line BL17U at the Shanghai Synchrotron Radiation Facility (SSRF). For each crystal, a total of 180 images were collected at a crystal-to-detector distance of 200 mm, with 0.4 s exposure for each 1° oscillation frame. Intensity data were integrated and scaled using HKL2000 [31].

The structure of NX-5 was determined by molecular replacement using *PHASER* [32] and the structure of isomaltulose synthase SmuA from *P* *. rubrum* as the searching model (PDB ID: 3GBD). The complex structures of E295Q/sucrose and D241A/glucose were solved by molecular replacement using *PHASER* [32] and the NX-5 structure as the searching model. Manual model building was performed with *COOT* [33]. Multiple rounds of refinement were carried out with *REFMAC5* [34] and *PHENIX* [35]. The overall qualities of the final models were assessed by *MolProbity* [36] and *PROCHECK* [37]. All graphics were generated using *PyMOL* [38] while the surface electrostatic potential of NX-5 was calculated by *DelPhi* [39].

Coordinates of NX-5, the E295Q/sucrose complex and the E241A/glucose complex have been deposited in the Protein Data Bank under the accession numbers of 4HOW, 4HPH and 4HOZ.

### Molecular docking and molecular dynamics simulations

Docking of fructofuranose and fructopyranose into the active site of the D241A/glucose complex was considered to mimic the transition state analogs of isomaltulose and trehalulose, respectively. Molecular docking was performed using the GOLD program (version 5.1, released by Cambridge Crystal Data Center) [40]. The binding site was defined by residues within 10 Å of the D-glucose molecule. For each docking, 30 conformations were kept while other parameters were set as default. The best-scored conformation was selected as that with the root-mean-square deviation (RMSD) value of less than 2.0 Å. A scoring function of GoldScore was used to evaluate the binding affinity between ligands and the enzyme.

MD simulations were performed in water from explicit models for 5 ns. The force field parameters applied to the small-molecule ligand were prepared by applying the Antechamber module in AMBER 9 [41]. Atomic partial charges on the small-molecule ligand were derived with the RESP method [42] based on the HF/6-31G* computation results given by the Gaussian software (version 03, released by Gaussian Inc) [43]. Atoms on the protein were assigned the PARM99 template charges implemented in AMBER, and all ionizable residues were set at the default protonation states at a neutral pH. The complex structure was placed in a box of TIP3P water molecules with a margin of 10 Å along each dimension. An appropriate number of counterions were added to neutralize the whole system. After these preparations, the complex structure was first minimized for 5000 steps with the protein and transition analogs under a restraint. The structure was further relaxed for 5000 steps without restraint. After that, the systems were gradually heated up using the Berendsen algorithm [44] from 0 K to 300 K in 100 ps. MD simulations were performed for each complex at a constant temperature of 300 K and a constant pressure of 1 atm for 5 ns. During the heating and equilibrating processes, the active residues around the binding site were kept restrained. The restraint force constant was set as 10.0 kcal/mol·Å^2^. Electrostatic interactions were calculated using the PME algorithm [45]. The distance cutoff of non-bonded interactions was set as 12 Å. The SHAKE algorithm [46] was applied to fix the lengths of all chemical bonds connecting the hydrogen atoms.

The complex structure of NX-5 mutant R335H/R336T/K337I/D338P (namely Mut1) and D-glucose was modeled by Discovery Studio software (version 3.5, Accerlys Inc.) using the D241A/glucose complex as a template. Residues Arg335, Arg336, Lys337 and Asp338 were mutated into histidine, threonine, isoleucine and proline by using the Protein Edit Module. The modeled complex was further subjected to 5 ns MD simulations in a similar way as described above.

## Results and Discussion

### Overall structure of isomaltulose synthase NX-5

The isomaltulose synthase NX-5 used for the crystallographic study contained residues 1-600 and a C-terminal His-tag derived from the pET-22b expression vector. The calculated molecular weight for the monomeric protein is 70.84 kDa, slightly larger than the observed mass (66.5 kDa) by SDS-PAGE analysis. Compared to our previous report [29], the new recombinant protein does not harbor the N-terminal peptide sequence derived from the expression vector.

**Figure 2:**
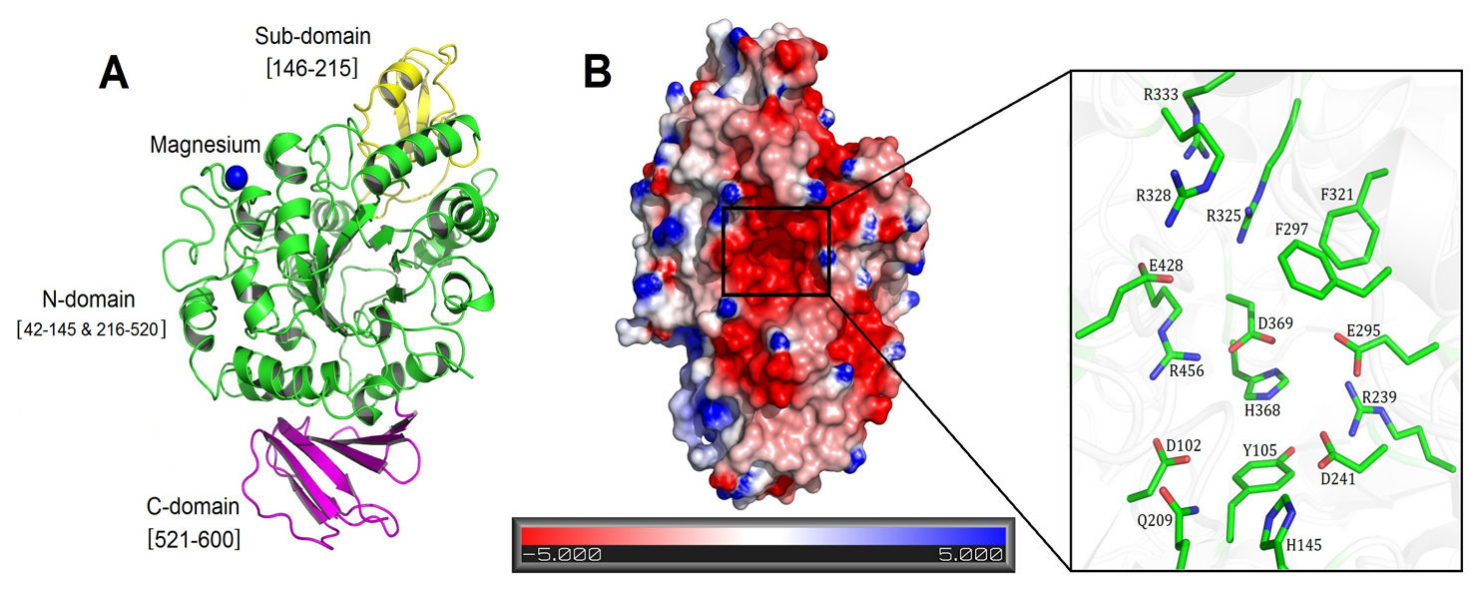
The crystal structure of native NX-5. (A) The overall structure of native NX-5 protein. The N-domain, Sub-domain and the C-domain are colored in green, yellow and magenta, respectively. The magnesium ion is depicted as a blue sphere. (B) The charge state of the solvent-accessible surface of NX-5 is predominantly basic. The surface was colored according to the electrostatic potential of the molecule. A zoomed view of the key residues in the active site pocket is also shown.

Crystals of NX-5 diffracted to 1.70 Å resolution and belonged to the space group *P*2 _1_2 _1_2_1_, with unit-cell parameters a = 58.9 Å, b = 81.3 Å, c = 138.0 Å (Table 1). There is one molecule in the asymmetric unit of the crystal lattice. The final model included residues 42-600 of NX-5 and two glycerol molecules while the electron density for the C-terminal His-tag was not visible. Similar to other GH13 family enzymes, the NX-5 molecule is composed of three domains (Figure 2A): the N-domain (residues 42-145 and 216-520), the sub-domain (residues 146-215), and the C-domain (residues 521-600). Among them, the N-domain is sandwiched between the sub-domain and C-domain and contains an imperfect (α/β)_8_ barrel with eight α-helices and seven β-strands. It includes all the residues involved in substrate binding and catalysis, demonstrating its functional importance which is similar to the corresponding parts of PalI and MutB [12,13]. The sub-domain consists of one α-helix (α4) and three alternating β-strands (β6, β7, and β8). Residues Phe185, Phe186, Phe205, and Gln209 in this region constitute the sidewall of the active pocket. The C-domain is composed of two antiparallel β-sheets, a larger five β-stranded (β14, β15, β16, β18 and β20) sheet and a smaller two β-stranded (β17 and β19) sheet. The C-domain interacts with the N-domain via the formation of salt bridges and hydrogen bonds (Figure S1 in File S1).

**Table 1:** Data collection and refinement statistics^**a**^.

	Wild-type	E295Q/sucrose	D241A/glucose
PDB ID	4HOW	4HPH	4HOZ
**Data Collection**		
Resolution range (Å)	45.1-1.70	35.0-1.70	31.8-2.00
	(1.76-1.70)	(1.76-1.70)	(2.07-2.00)
Space group	*P*2_1_2_1_2_1_	*P*2_1_2_1_2_1_	*P*2_1_2_1_2_1_
*a*, *b*, *c* (Å)	58.9, 81.3, 138.0	58.6, 81.1, 138.4	58.7, 82.5, 137.9
Redundancy	4.4 (3.0)	6.0 (5.9)	6.6 (6.3)
Completeness (%)	97.8 (84.0)	99.2 (99.0)	98.5 (97.0)
No. of reflections (unique)	609771 (72135)	434369 (72577)	299859 (45383)
*I/σ*	20.4 (5.0)	13.0 (3.7)	18.6 (2.8)
*R*_merge_^b^	0.075 (0.509)	0.112 (0.572)	0.084 (0.491)
Molecules in the AU	1	1	1
**Refinement**			
*R*_work_^c^/*R*_free_^d^	0.156/0.188	0.167/0.198	0.147/0.197
Average *B* factor (Å^2^)	20.0	19.4	26.2
RMSD bonds (Å)	0.006	0.006	0.008
RMSD angles (°)	1.04	1.05	1.03
Ramachandran statistics^e^	97.16%, 2.84%, 0%	97.16%, 2.84%, 0%	97.15%, 2.85%, 0%

a Values in parentheses are for the highest resolution shell.

b $R_{\text{merge}}=\sum_{\text{hkl}}\sum_{\text{i}}|I(hkl;i)–\;<I(hkl)>|/\sum_{\text{hkl}}\sum_{\text{i}}I(hkl;i)\;$, where *I(*hkl;i) is the intensity of an individual measurement of a reflection and <I(hkl)> is the average intensity of that reflection.

c $R_{\text{work}}=\;[\sum |\left(F_{\text{obs}}\right)\;–k\left(F_{\text{calc}}\right)]/\sum \left|\left({\text{F}}_{\text{obs}}\right)\right|],$ where *k* is a scale factor and the test set has been removed.

d *R*_free_ is defined as the *R*_work_ calculated for 9% of the X-ray data selected randomly, and excluded from refinement.

e Ramachandran statistics indicate the fraction of residues in the most favored, allowed, and disallowed regions of the Ramachandran diagram, respectively.

A hexa-coordinate magnesium ion was found to interact with five residues (Asp63, Asn65, Asp67, Ile69, and Asp71) highly conserved in all known SIases (Figure S2 in File S1). Because there was no magnesium containing reagents used in the crystallization experiment, the metal ion must be derived from the medium used for the *E. coli* cell culture. In the NX-5 structure, the magnesium ion was about 23 Å away from the active site pocket, suggesting its function may be structural rather than catalytic. As reported earlier, a calcium ion was found in the corresponding position of the MutB structure whereas water molecules were observed in the structures of SmuA and PalI [12,24].

### The structural features of the active site

The active site of NX-5 is located in a pocket that is approximately 25 Å deep from the negatively charged surface (Figure 2B). Several key residues conserved among NX-5, PalI, MutB, and SmuA are involved in substrate binding, enzymatic hydrolysis, and disaccharide formation. Among them, residues Asp102, His145, Arg239, His368, Asp369, Glu428, and Arg456 are responsible for sucrose binding and residues Asp241 (the nucleophile) and Glu295 (the acid catalyst) are crucial for sucrose hydrolysis as described previously for PalI, MutB, and SmuA (Figure 2B, Figure S3 in File S1) [12,13,24]. The active site pocket is stabilized by the salt bridge interactions between Arg239-Asp241, Asp102-Arg456 and Asp369-Arg456. Residues Arg325 and Arg328 are located in the^325^RLDRD^329^ motif and play roles in the product specificity. Site-directed mutation of Arg325 (PalI) and Arg328 (SmuA) reduced isomaltulose production and slightly increased trehalulose production [15,26]. Mutations of Phe164 to leucine and Arg284 to cysteine in MutB resulted in the active-site opening, which allowed the mutant enzymes favor hydrolytic activities [25].

Similar to the function of the corresponding residues Phe256/Phe270 and Phe280/Phe294 in MutB/SmuA [13,24], Phe297 and Phe321 in NX-5 form an aromatic clamp near the entrance of the active pocket. These two residues are conserved in NX-5 and MutB but their side chain conformations differ. As shown in Figure S4 in File S1, Phe321 and Phe297 in NX-5 are closer to the catalytic^325^RLDRD^329^ motif and residue Glu295. Replacement of either residue by alanine resulted in retention of the sucrose hydrolytic activity but disrupted its isomerization function (Table 2). Interestingly, the F297A mutant of NX-5 gained the ability to synthesize a novel α-arbutin derivative [47]. This result is consistent with the conclusion that Phe256 and Phe280 in MutB were essential for product specificity [13,25]. Gorl et al. observed a similar result that mutagenesis of Phe297 and Phe321 in SmuA abolished the isomaltulose synthesis activity [48].

**Table 2:** Sugar composition of the isomerization products from sucrose and the general catalytic characteristics of different NX-5 mutants.

Enzyme	Specific activity (U/mg)	Glucose and fructose (%)	Isomaltulose (%)	Trehalulose (%)	Product ratio
Wild-type	401.0±0.3	4.2±0.2	83.2±0.3	12.6±0.3	6.6:1.0
F297A	77.2±0.5	100	0	0	—
F321A	98.0±0.5	100	0	0	—
R325D	188.4±0.5	54.1±0.3	20.4±0.1	25.5±0.2	0.8:1.0
R328D	237.9±0.4	17.4±0.2	60.9±0.3	21.7±0.2	2.8:1.0
ΔGlu332	186.0±0.2	8.3±0.2	79.4±0.2	12.3±0.1	6.5:1.0
Mut1^a^	205.0±0.3	7.4±0.2	41.7±0.2	50.9±0.2	0.8:1.0
Mut2^b^	387.7±0.5	6.0±0.1	46.6±0.2	47.4±0.3	1.0:1.0

The product ratio indicates the proportion of isomaltulose to trehalulose in the final reaction products.

a Mut1: R335H/R336T/K337I/D338P

b Mut2: A330L/ΔGlu332/R335H/R336T/K337I/D338P/W339R

### The complex structures of E295Q/sucrose and D241A/glucose

**Figure 3:**
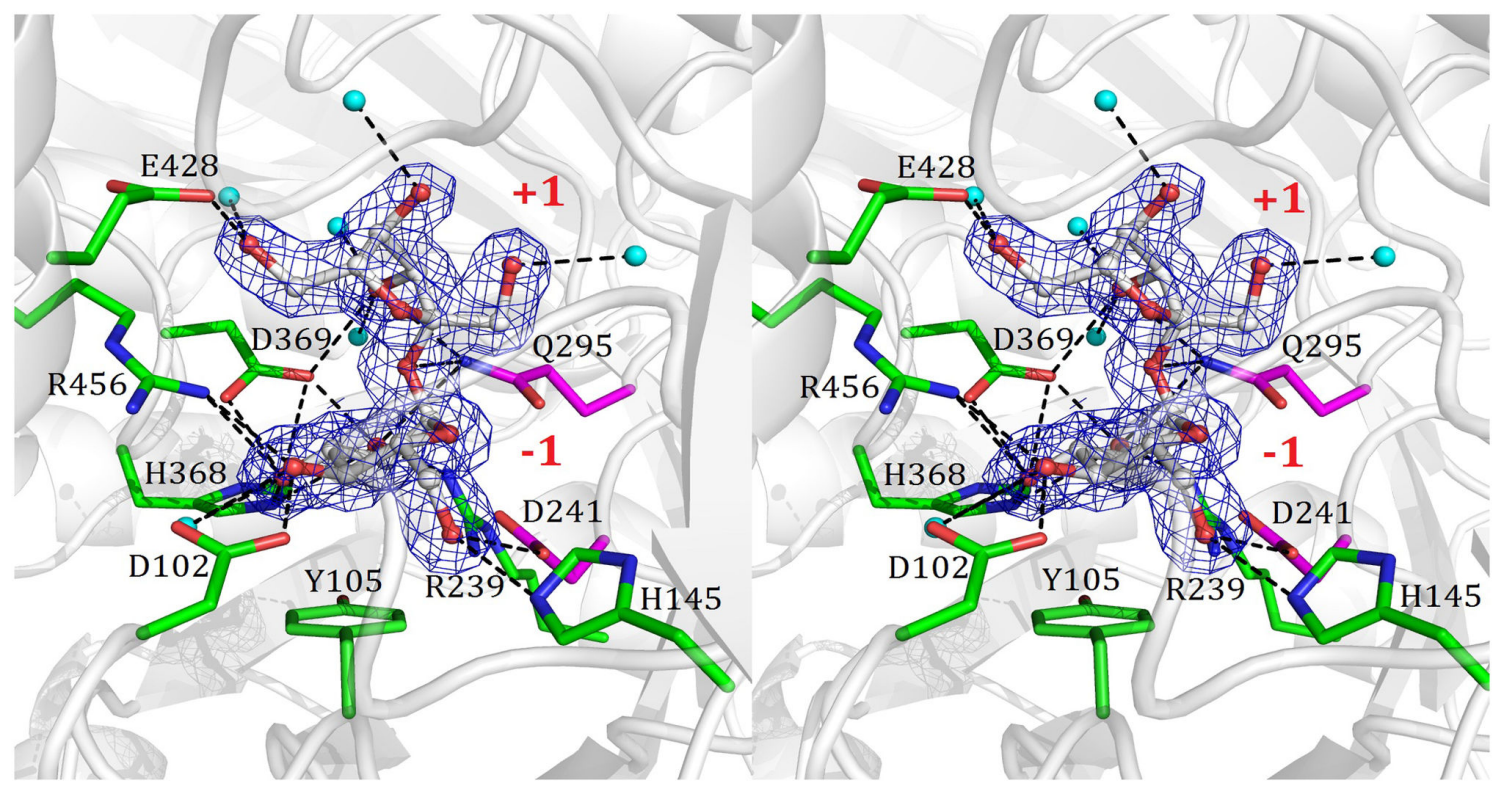
A stereo view of the binding mode between sucrose and NX-5 protein in the E295Q/sucrose complex. The binding mode of sucrose in the E295Q/sucrose complex is shown in a stereoscopic view. The 2Fo-Fc electron density maps are contoured at 1σ. The water molecule is highlighted as a cyan ball and the hydrogen bonds are depicted as dashed lines.

To investigate the mechanism of substrate binding and sucrose isomerization, we determined the crystal structure of the inactive E295Q mutant in complex with the substrate sucrose at 1.70 Å resolution. In the structure, the sucrose molecule was deeply bound in the active site with the glucosyl ring at subsite -1 and fructosyl moiety at subsite +1 (Figure 3). The glucosyl ring of sucrose interacts with a water molecule and eight residues of NX-5 (Asp102, His145, Arg239, Asp241, Gln295, His368, Asp369, and Arg456) via a hydrogen-bond network as well as through π-π stacking with Tyr105 and Phe205. The fructosyl moiety of sucrose binds to NX-5 through direct hydrogen bonds with Gln295, Asp369 and Glu428, hydrophobic interactions with Phe297, and hydrogen bonds between 1’-OH, 3’-OH, 4’-OH, 6’-OH and four water molecules. Therefore, the fructosyl moiety of sucrose has fewer interactions with the active site pocket, which should allow it to exit easily after sucrose hydrolysis and undergo tautomerization resulting in trehalulose production.

Inspired by the work from Ravaud et al. [13], we obtained the crystals of the D241A**/**glucose complex by co-crystallizing the D241A mutant with sucrose. As mentioned above, Asp241 is the key nucleophile residue involved in the catalysis of the glycosidic bond cleavage. In the 2.00 Å structure, clear electron density of D-glucose rather than sucrose was identified in the active site (Figure S5A in File S1). This demonstrated that replacement of Asp241 with an alanine residue did not totally disrupt the hydrolytic activity of NX-5, as observed in the complex structure of MutB D200A-glucose [13]. Moreover, the D241A**/**glucose complex structure may represent NX-5 in an intermediate state of the sucrose isomerization process and could be used for molecular dynamics (MD) calculations. The structures of the D241A**/**glucose and E295Q**/**sucrose complexes are highly similar with a RMSD value of 0.14 Å, indicating that little conformational change happens after sucrose hydrolysis (Figure S5B in File S1).

Superimposition of 15 residues (Asp102, His145, Gln209, Arg239, Ala241, Glu295, Phe297, Phe321, Arg325, Arg328, Arg333, His368, Asp369, Glu428 and Arg456) in the substrate-binding pocket of the native NX-5, the E295Q/sucrose complex and the D241A/glucose complex show subtle structural changes in the active site pocket with an RMSD of 0.20 Å between the native NX-5 and the E295Q/sucrose complex (Figure S6A in File S1) and an RMSD of 0.15 Å between the native NX-5 and the D241A/glucose complex (Figure S6B in File S1). These residues are highly conserved among NX-5, PalI, SmuA, and MutB. Upon sucrose binding, the phenyl rings of Phe297 and Phe321 were forced to move away from the substrate. After sucrose hydrolysis and the exiting of fructose, their side chains bounced back to the original position in the active site pocket. Substrate binding also pushed the side chain of Arg325 towards Arg328.

### Structural comparison of isomaltulose synthase NX-5 and trehalulose synthase MutB

A structural comparison between NX-5 and other reported SIases such as SmuA, PalI and MutB as well as a GH13 family oligo-1,6-glucosidase (OGL) from *Bacillus cereus* showed that they were highly homologous (Figure S7 in File S1). The RMSD between the Cα atoms of NX-5 and SmuA (3GBD), PalI (1M53), MutB (2PWH), and OGL (1UOK) were 0.44, 0.80, 0.72, and 1.49 Å, respectively. The results indicated that the subtle conformational variance in the active pocket of isomaltulose synthase and trehalulose synthase might play an essential role in the product specificity of sucrose isomerization.

**Figure 4:**
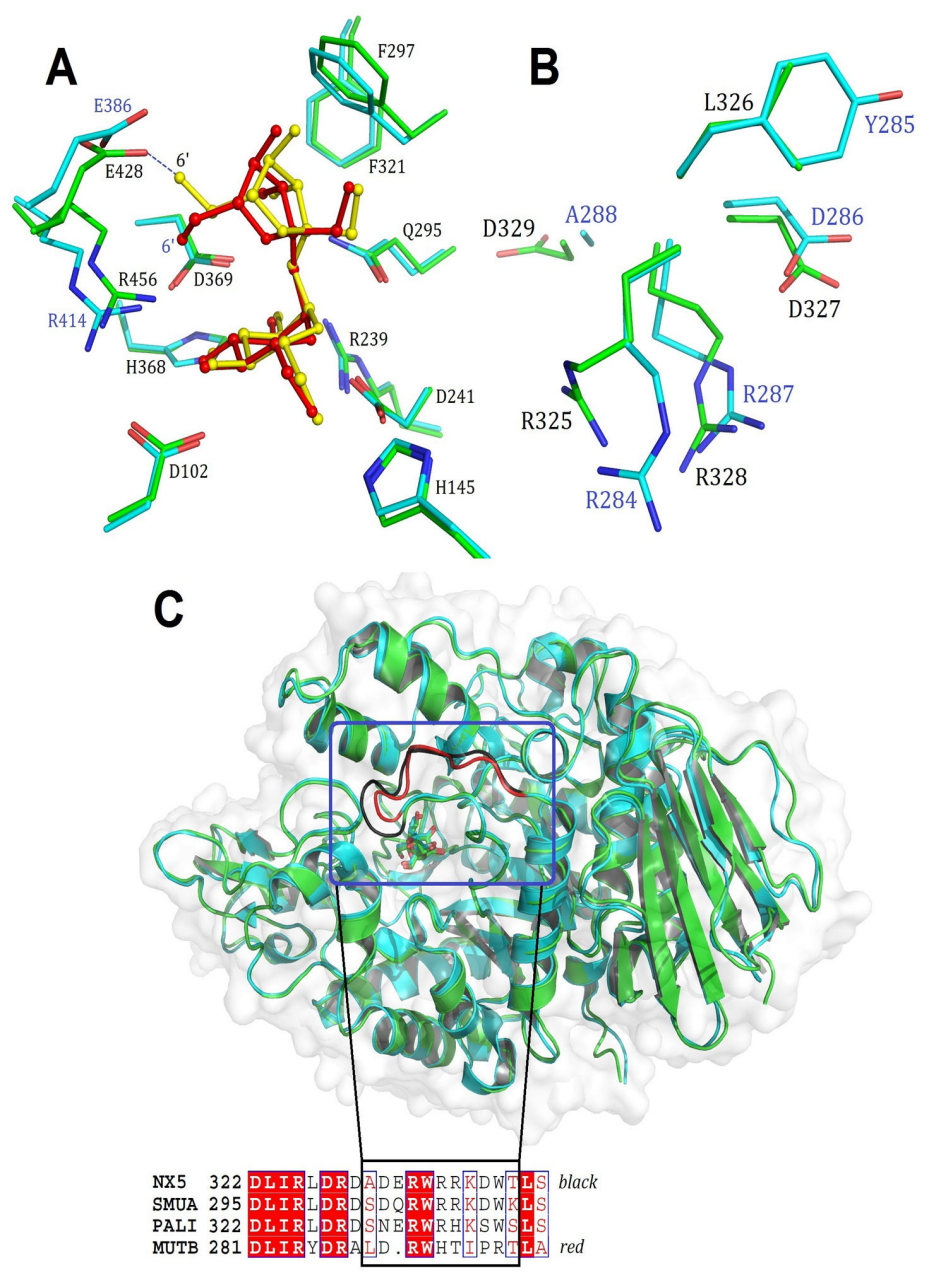
Structural difference between NX-5/sucrose and MutB/sucrose. (A) Structural alignment of the active site residues between NX-5/sucrose (PDB ID: 4HPH, green) and MutB/sucrose (2PWE, cyan). The sucrose molecule in NX-5 is colored yellow, whereas that in MutB is colored red. Residues from NX-5 are labeled. (B) Structural comparison of^325^RLDRD^329^ in NX-5 (green) and^284^RYDRA^288^ (cyan) residues in MutB. (C) Structural and sequence comparison of loop^330-339^ (black) in NX-5 and the corresponding loop^289-297^ (red) of MutB.

To examine the catalytic differences between isomaltulose synthase and trehalulose synthase, we compared the structures of the E295Q/sucrose complex (as reported herein) and MutB/sucrose (PDB ID: 2PWE [13]). The RMSD value between the Cαs in the two structures was 0.70 Å. The orientation of seven conserved residues (Asp102, His145, Arg239, Asp241, Gln295, His368, and Asp369) of NX-5 involved in sucrose binding and hydrolysis was virtually identical to that of MutB (RMSD = 0.21 Å, Figure 4A). Residue Glu428 in NX-5 has a different side chain orientation from that of the corresponding residue Glu386 in MutB. Glu428 is involved in a 2.39 Å hydrogen bond to the 6’-OH of the fructosyl moiety, which induces the O6’-C6’ bond to rotate 98.9°.

The most prominent structural differences between NX-5 and MutB occur in the catalytic^325^RLDRD^329^ motif and its adjacent 10-residue loop (residues 330-339). As shown in Figure 4B, Arg325 of NX-5 and the corresponding Arg284 of MutB exhibited a significant difference in their side chain orientation. In earlier reports, Arg325 was deemed to be essential for the tautomerization from fructofuranose to fructopyranose and mutation of the corresponding residue in PalI and SmuA promoted trehalulose formation [15,26]. As expected, mutation of Arg325 to aspartic acid changed the product ratio of isomaltulose to trehalulose from 6.6:1.0 to 0.8:1.0 (Table 2). This mutation also greatly enhanced the production of glucose and fructose from 4.2% to 54.1%. When Arg328 was replaced by Asp, the product ratio of isomaltulose to trehalulose decreased to 2.8:1.0 (Table 2).

As shown in Figure 4C, the conformation of the 10-residue loop (residues 330-339) in NX-5 was remarkably different from that of the corresponding loop (residues 289-297) in MutB. The RMSD between the backbone atoms in the two loops was approximately 2.2 Å. One difference between them is that the loops from NX-5 and other ISs are one-residue longer than that of MutB (Figure 4C and Figure S3 in File S1). Another difference is that the loop of NX-5 contains the polar residues Arg335, Arg336, Lys337 and Asp338 whereas the corresponding part of MutB consists of non-polar residues including His292, Thr293, Ile294 and Pro295 (Figure S8 in File S1). The structural and sequence divergence between NX-5 and MutB revealed that this loop region may be essential for product specificity.

To investigate the function of loop^330-339^, we performed site-directed mutagenesis on NX-5 and measured the sucrose isomerase activity for each mutant. As shown in Table 2, the product ratio of the Glu332 deletion mutant was similar to that of the wild-type NX-5. When four polar residues of NX-5 were replaced by the corresponding residues of MutB, the product ratio of isomaltulose to trehalulose decreased to 0.8:1.0 while the specific activity was reduced to 51% (Mut1, Table 2). However, when the whole loop^330-339^ of NX-5 was replaced by loop^289-297^ of MutB, the product ratio of isomaltulose to trehalulose decreased to 1.0:1.0 but the total amount of isomaltulose plus trehalulose was almost identical to that from the wild-type NX-5 (Mut2, Table 2). Taken together, these results demonstrated that the 10-residue loop adjacent to the catalytic^325^RLDRD^329^ motif played an important role in product specificity control.

### Structural basis for the product specificity of NX-5

To elucidate the mechanism of the product specificity of NX-5, we used the GOLD program (version 5.1, released by the Cambridge Crystallographic Data Centre) [40] to dock fructofuranose or fructopyranose, a transition state analogue of isomaltulose or trehalulose into the active site of the D241A/glucose complex structure. The best-scored conformation from 30 candidates was then subjected to 5 ns molecular dynamics (MD) simulations. Both transition state analogs finally maintained stable-binding modes, as the RMSD curves were generally unaltered during the last 2 ns of the MD simulations (Figure S9 in File S1). As shown in Table 3, the free energy change of the transition state analog of trehalulose from the last 1 ns MD trajectory was -51.60 kcal/mol, 4.26 kcal/mol higher than that of isomaltulose (-55.86 kcal/mol). This result proved that isomaltulose was indeed the favored product of NX-5 catalysis.

**Table 3:** Free energy calculations for the isomaltulose and trehalulose transition state analogs in complex with NX-5.

Transition State Analogs	G_1_ (kcal/mol)	G_2_ (kcal/mol)	G_3_ (kcal/mol)	G_4_ (kcal/mol)	ΔG (kcal/mol)
fructofuranose	-11454.77	-11527.10	51.91	76.28	-55.86
fructopyranose	-11998.13	-12089.33	48.59	94.21	-51.60

ΔG was computed using the free energy of the NX-5/transition state complex minus the free energies of NX-5, D-glucose and fructofuranose/fructopyranose. ΔG values reflected the free energy changes after NX-5 bound with the transition state analog as described below.

G_1_: The free energy of the NX-5/transition state analog complex

G_2_: The free energy of NX-5

G_3_: The free energy of D-glucose

G_4_: The free energy of the ligand. Fructofuranose is the isomaltulose transition analog whereas the trehalulose transition analog is fructopyranose.

**Figure 5:**
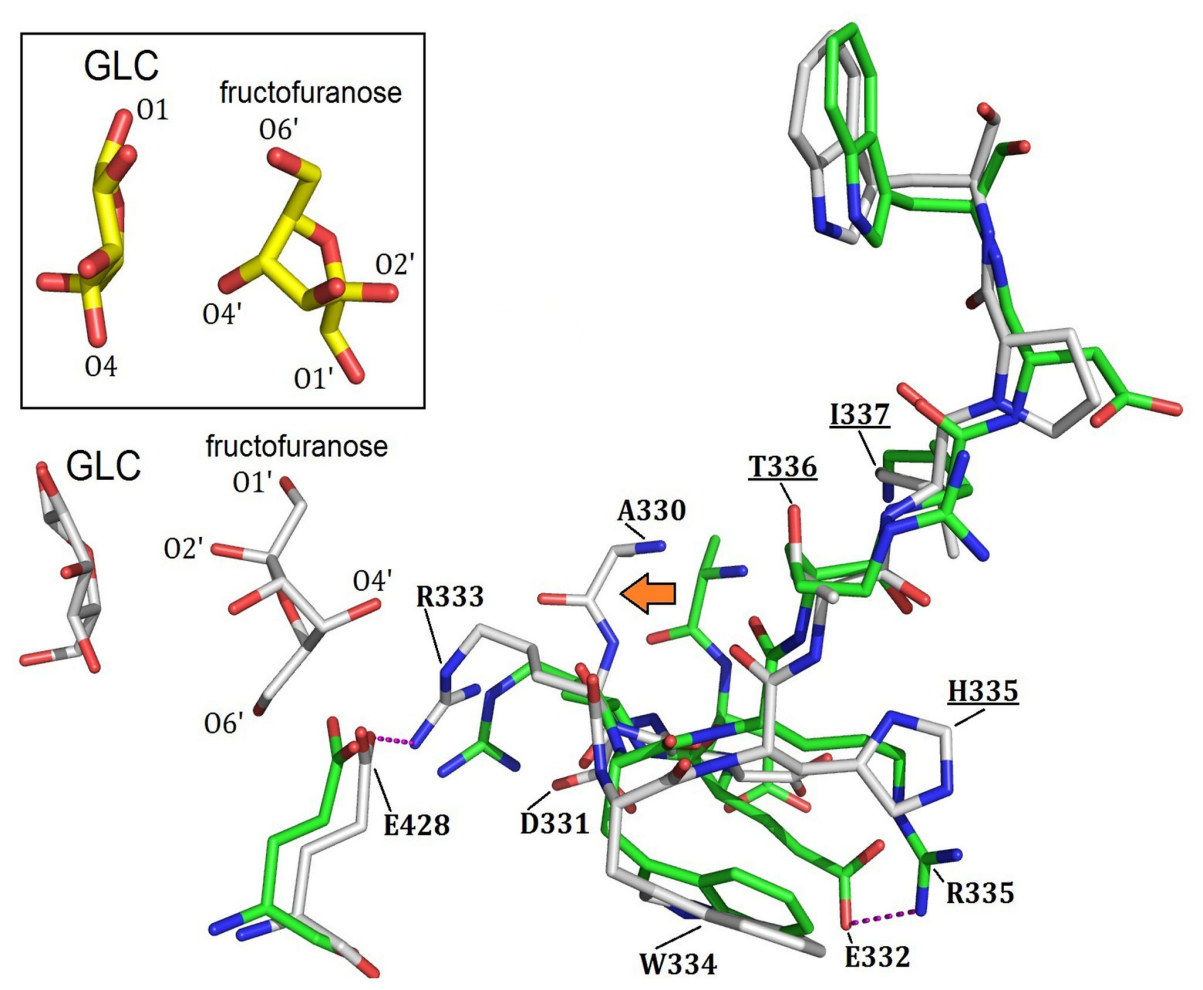
Structural comparison of the modeled D241A/glucose/fructofuranose complex (green) and the Mut1/glucose/fructofuranose (grey) complex. The D-glucose and fructofuranose in D241A are highlighted in a square.

We next modeled the complex structure of Mut1/glucose using Discovery Studio software (version 3.5, Accerlys Inc.) and carried out docking and MD simulations in a similar way as described above. By comparing the structures of wild-type NX-5 and the modeled structure of Mut1, we found the phenyl ring of Phe297 rotated towards D-glucose. The guanidino group of Arg333 and the carboxyl group of Glu428 also re-oriented to form hydrogen bond interactions. Replacement of Arg335 by histidine destroyed the hydrogen bond interactions between Arg335 and Glu332. As a result, the side chain of Glu332 shifted 3.3 Å towards the left together with Ala330 and Asp331 (Figure 5). This change further rendered the guanidino group of Arg333 to move left to form a hydrogen bond with Glu428 and decreased the possibility of hydrogen bonding between Arg333 and the 1’-OH and 2’-OH groups of fructofuranose. These changes were important as it led to fructofuranose adopting a conformation similar to the isomaltulose transition state as observed in D241A (Figure 5). The conformation of fructofuranose was therefore changed and decreased the possibility of isomaltulose formation.

## Conclusions

This work revealed for the first time the enzyme/substrate complex structure of an isomaltulose synthase, NX-5. Comparison between the NX-5/sucrose and MutB/sucrose complex structures highlighted a unique loop that participated in governing the product specificity besides the known catalytic RXDRX motif. Our molecular dynamics simulation studies demonstrated the influence of loop^330-339^ on isomaltulose synthesis, as the mutant R335H/R336T/K337I/D338P did not have hydrogen bonds between Glu332 and Arg335, which resulted in formation of a new hydrogen bond between Arg333 and Glu428. These structural perturbations weakened the conformational stability of the fructofuranose 1’-OH and 2’-OH groups, which finally altered the product specificity. Collectively, these results indicated the importance of loop^330-339^ in the product specificity control of the SIase NX-5 and provided structural information helpful for elucidating substrate binding and the potential catalytic mechanism of NX-5.

## Supporting Information

File S1 (DOC)

## References

[1] Kawai K, Okuda Y, Yamashita K (1985) Changes in blood glucose and insulin after an oral palatinose administration in normal subjects. Endocrinol Jpn 32: 933-936. doi:10.1507/endocrj1954.32.933. PubMed: 3914416. 3914416 10.1507/endocrj1954.32.933

[2] Hamada S (2002) Role of sweeteners in the etiology and prevention of dental caries. Pure Appl Chem 74: 1293-1300. doi:10.1351/pac200274071293.

[3] Matsukubo T, Takazoe I (2006) Sucrose substitutes and their role in caries prevention. Int Dent J 56: 119-130. PubMed: 16826877. 16826877 10.1111/j.1875-595x.2006.tb00083.x

[4] Lina BA, Jonker D, Kozianowski G (2002) Isomaltulose (palatinose): a review of biological and toxicological studies. Food Chem Toxicol 40: 1375-1381. doi:10.1016/S0278-6915(02)00105-9. PubMed: 12387299. 12387299 10.1016/s0278-6915(02)00105-9

[5] Takazoe I (1989) Palatinose-an isomeric alternative to sucrose. In Grenby TH, Progress in sweeteners. Barking, UK: Elsevier pp. 143-167.

[6] Baer A (1989) Significance and promotion of sugar substitution for the prevention of dental caries. Lebensm Wiss Technol 22: 46-53.

[7] Lichtenthaler FW, Peters S (2004) Carbohydrates as green raw materials for the chemical industry. Compt Rend Chim 7: 65-90. doi:10.1016/j.crci.2004.02.002.

[8] Cheetham PSJ, Imber CE, Isherwood J (1982) The formation of isomaltulose by immobilized *Erwinia* *rhapontici* . Nature 299: 628-631. doi:10.1038/299628a0.

[9] Cheetham PS (1984) The extraction and mechanism of a novel isomaltulose-synthesizing enzyme from *Erwinia* *rhapontici* . Biochem J 220: 213-220. PubMed: 6743261. 6743261 10.1042/bj2200213 PMC1153612

[10] Huang JH, Hsu LH, Su YC (1998) Conversion of sucrose to isomaltulose by *Klebsiella* *planticola* CCRC 19112. J Ind Microbiol Biotechnol 21: 22-27. doi:10.1038/sj.jim.2900552.

[11] Veronese T, Perlot P (1998) Proposition for the biochemical mechanism occurring in the sucrose isomerase active site. FEBS Lett 441(3): 348-352. doi:10.1016/S0014-5793(98)01582-8. PubMed: 9891968. 9891968 10.1016/s0014-5793(98)01582-8

[12] Zhang D, Li N, Lok SM, Zhang LH, Swaminathan K (2003) Isomaltulose synthase (PalI) of *Klebsiella* sp. LX3. Crystal structure and implication of mechanism. J Biol Chem 278: 35428-35434. doi:10.1074/jbc.M302616200. PubMed: 12819210. 12819210 10.1074/jbc.M302616200

[13] Ravaud S, Robert X, Watzlawick H, Haser R, Mattes R et al. (2007) Trehalulose synthase native and carbohydrate complexed structures provide insights into sucrose isomerization. J Biol Chem 282: 28126-28136. doi:10.1074/jbc.M704515200. PubMed: 17597061. 17597061 10.1074/jbc.M704515200

[14] Zhang D, Li X, Zhang LH (2002) Isomaltulose synthase from *Klebsiella* sp. strain LX3: gene cloning and characterization and engineering of thermostability. Appl Environ Microbiol 68: 2676-2682. doi:10.1128/AEM.68.6.2676-2682.2002. PubMed: 12039719. 12039719 10.1128/AEM.68.6.2676-2682.2002 PMC123955

[15] Lee HC, Kim JH, Kim SY, Lee JK (2008) Isomaltose production by modification of the fructose-binding site on the basis of the predicted structure of sucrose isomerase from "*Protaminobacter* *rubrum*". Appl Environ Microbiol 74: 5183-5194. doi:10.1128/AEM.00181-08. PubMed: 18552181. 18552181 10.1128/AEM.00181-08 PMC2519274

[16] Veronese T, Perlot P (1999) Mechanism of sucrose conversion by the sucrose isomerase of *Serratia* *plymuthica* ATCC 15928. Enzyme Microb Technol 24: 263-269. doi:10.1016/S0141-0229(98)00115-X.

[17] McAllister M, Kelly CT, Doyle E, Fogarty WM (1990) The isomaltulose synthesizing enzyme of *Serratia* *plymuthica* . Biotechnol Lett 12: 667-672. doi:10.1007/BF01088191.

[18] Wu L, Birch RG (2004) Characterization of *Pantoea* *dispersa* UQ68J: producer of a highly efficient sucrose isomerase for isomaltulose biosynthesis. J Appl Microbiol 97: 93-103. doi:10.1111/j.1365-2672.2004.02274.x. PubMed: 15186446. 15186446 10.1111/j.1365-2672.2004.02274.x

[19] Cho MH, Park SE, Lim JK, Kim JS, Kim JH et al. (2007) Conversion of sucrose into isomaltulose by *Enterobacter* sp. FMB1, an isomaltulose-producing microorganism isolated from traditional Korean food. Biotechnol Lett 29: 453-458. doi:10.1007/s10529-006-9257-6. PubMed: 17160347. 17160347 10.1007/s10529-006-9257-6

[20] Nagai-Miyata Y, Tsuyuki K, Sugitani T, Ebashi T, Nakajima Y (1993) Isolation and characterization of a trehalulose-producing strain of *Agrobacterium* . Biosci Biotechnol Biochem 57: 2049-2053. doi:10.1271/bbb.57.2049.

[21] Nagai Y, Sugitani T, Tsuyuki K (1994) Characterization of alpha-glucosyltransferase from *Pseudomonas* *mesoacidophila* MX-45. Biosci Biotechnol Biochem 58: 1789-1793. doi:10.1271/bbb.58.1789. PubMed: 7765505. 7765505 10.1271/bbb.58.1789

[22] Miyata Y, Sugitani T, Tsuyuki K, Ebashi T, Nakajima Y (1992) Isolation and characterization of *Pseudomonas* *mesoacidophila* producing trehalulose. Biosci Biotechnol Biochem 56: 1680-1681. doi:10.1271/bbb.56.1680.

[23] Goulter KC, Hashimi SM, Birch RG (2012) Microbial sucrose isomerases: Producing organisms, genes and enzymes. Enzyme Microb Technol 50: 57-64. doi:10.1016/j.enzmictec.2011.09.011. PubMed: 22133441. 22133441 10.1016/j.enzmictec.2011.09.011

[24] Ravaud S, Robert X, Watzlawick H, Haser R, Mattes R et al. (2009) Structural determinants of product specificity of sucrose isomerases. FEBS Lett 583: 1964-1968. doi:10.1016/j.febslet.2009.05.002. PubMed: 19427862. 19427862 10.1016/j.febslet.2009.05.002

[25] Lipski A, Watzlawick H, Ravaud S et al. (2013) Mutations inducing an active-site aperture in *Rhizobium* sp. sucrose isomerase confer hydrolytic activity. Acta Crystallogr D Biol Crystallogr 69: 298-307. doi:10.1107/S0907444912045532. PubMed: 23385465. 23385465 10.1107/S0907444912045532

[26] Zhang D, Li N, Swaminathan K, Zhang LH (2003) A motif rich in charged residues determines product specificity in isomaltulose synthase. FEBS Lett 534: 151-155. doi:10.1016/S0014-5793(02)03835-8. PubMed: 12527377. 12527377 10.1016/s0014-5793(02)03835-8

[27] Aroonnual A, Nihira T, Seki T, Panbangred W (2007) Role of several key residues in the catalytic activity of sucrose isomerase from *Klebsiella* *pneumoniae* NK33-98-8. Enzyme Microb Technol 40: 1221-1227. doi:10.1016/j.enzmictec.2006.09.011.

[28] Ren B, Li S, Xu H, Feng XH, Cai H et al. (2011) Purification and characterization of a highly selective sucrose isomerase from *Erwinia* *rhapontici* NX-5. Bioprocess Biosyst Eng 34: 629-637. doi:10.1007/s00449-010-0512-9. PubMed: 21229265. 21229265 10.1007/s00449-010-0512-9

[29] Li S, Cai H, Qing Y, Ren B, Xu H et al. (2011) Cloning and characterization of a sucrose isomerase from *Erwinia* *rhapontici* NX-5 for isomaltulose hyperproduction. Appl Biochem Biotechnol 163: 52-63. doi:10.1007/s12010-010-9015-z. PubMed: 20589449. 20589449 10.1007/s12010-010-9015-z

[30] Bradford MM (1976) A rapid and sensitive method for the quantitation of microgram quantities of protein utilizing the principle of protein-dye binding. Anal Biochem 72: 248-254. doi:10.1016/0003-2697(76)90527-3. PubMed: 942051. 942051 10.1016/0003-2697(76)90527-3

[31] Otwinowski Z, Minor W (1997) Processing of X-ray diffraction data collected in oscillation mode. Methods Enzymol 276: 307-326. doi:10.1016/S0076-6879(97)76066-X. 10.1016/S0076-6879(97)76066-X 27754618

[32] Mccoy AJ, Grosse-Kunstleve RW, Adams PD, Winn MD, Storoni LC et al. (2007) Phaser crystallographic software. J Appl Crystallogr 40: 658-674. doi:10.1107/S0021889807021206. PubMed: 19461840. 19461840 10.1107/S0021889807021206 PMC2483472

[33] Emsley P, Lohkamp B, Scott WG, Cowtan K (2010) Features and development of Coot. Acta Crystallogr D Biol Crystallogr 66: 486-501. doi:10.1107/S0907444910007493. PubMed: 20383002. 20383002 10.1107/S0907444910007493 PMC2852313

[34] Vagin AA, Steiner RA, Lebedev AA, Potterton L, McNicholas S et al. (2004) REFMAC5 dictionary: organization of prior chemical knowledge and guidelines for its use. Acta Crystallogr D Biol Crystallogr 60: 2184-2195. doi:10.1107/S0907444904023510. PubMed: 15572771. 15572771 10.1107/S0907444904023510

[35] Adams PD, Afonine PV, Bunkóczi G, Chen VB, Davis IW et al. (2010) PHENIX: a comprehensive Python-based system for macromolecular structure solution. Acta Crystallogr D Biol Crystallogr 66: 213-221. doi:10.1107/S0907444909052925. PubMed: 20124702. 20124702 10.1107/S0907444909052925 PMC2815670

[36] Chen VB, Arendall WB, Headd JJ, Keedy DA, Immormino RM et al. (2010) MolProbity: all-atom structure validation for macromolecular crystallography. Acta Crystallogr D Biol Crystallogr 66: 12-21. doi:10.1107/S1744309109042018. PubMed: 20057044. 20057044 10.1107/S0907444909042073 PMC2803126

[37] Laskowski RA, Macarthur MW, Moss DS, Thornton JM (1993) Procheck - a program to check the stereochemical quality of protein structures. J Appl Crystallogr 26: 283-291. doi:10.1107/S0021889892009944.

[38] Delano WL (2002) The PyMOL Molecular Graphics System. San Carlos, CA: DeLano Scientific.

[39] Li L, Li C, Sarkar S, Zhang J, Witham S et al. (2012) DelPhi: a comprehensive suite for DelPhi software and associated resources. BMC Biophys 5: 9. doi:10.1186/2046-1682-5-9. PubMed: 22583952. 22583952 10.1186/2046-1682-5-9 PMC3463482

[40] Jones G, Willett P, Glen RC, Leach AR, Taylor R (1997) Development and validation of a genetic algorithm for flexible docking. J Mol Biol 267: 727-748. doi:10.1006/jmbi.1996.0897. PubMed: 9126849. 9126849 10.1006/jmbi.1996.0897

[41] Case DA, Darden TA, Cheatham TE et al. (2006) AMBER 9. University of California San Francisco.

[42] Bayly CI, Cieplak P, Cornell WD, Kollman PA (1993) A well-behaved electrostatic potential based method using charge restraints for deriving atomic charges - the RESP model. J Phys Chem 97: 10269-10280. doi:10.1021/j100142a004.

[43] Frisch MJ, Head-Gordon M, Pople JA (1990) Directed analytic SCF second derivatives and electric field properties. J Chem Phys 141: 189-196.

[44] Berendsen HJC, Postma JPM, Vangunsteren WF, Dinola A, Haak JR (1984) Molecular-dynamics with coupling to an external bath. J Chem Phys 81: 3684-3690. doi:10.1063/1.448118.

[45] Darden T, York D, Pedersen L (1993) Particle mesh Ewald - an N.log(N) method for Ewald sums in large systems. J Chem Phys 98: 10089-10092. doi:10.1063/1.464397.

[46] Miyamoto S, Kollman PA (1992) Settle - an analytical version of the shake and rattle algorithm for rigid water models. J Comput Chem 13: 952-962. doi:10.1002/jcc.540130805.

[47] Zhou X, Zheng Y, Wei X, Yang K, Yang X et al. (2011) Sucrose isomerase and its mutants from *Erwinia* *rhapontici* can synthesise α-arbutin. Protein Pept Lett 18: 1028-1034. doi:10.2174/092986611796378774. PubMed: 21592077. 21592077 10.2174/092986611796378774

[48] Gorl J, Timm M, Siebel J (2012) Mechanism-oriented redesign of an isomaltulose synthase to an isomelezitose synthase by site-directed mutagenesis.. Chembiochem 13: 149-156. doi:10.1002/201100576. 22124943 10.1002/cbic.201100576

