# Supplementary material for: The Structural Basis of Erwinia rhapontici Isomaltulose Synthase
Source: PLoS One. 2013 Sep 19;8(9):e74788. doi: 10.1371/journal.pone.0074788 (PMC3777934; doi:10.1371/journal.pone.0074788)
Supplement: File S1 — (DOC) [file pone.0074788.s001.doc]

**Supporting Information**

The structural basis of *Erwinia rhapontici* isomaltulose synthase

**Zheng Xu^1, 2^, Sha Li^1^, Jie Li^2^, Yan Li^2^, Xiaohai Feng^1^, Renxiao Wang^2^, Hong Xu^1^*, Jiahai Zhou^2^***

^1^ *State Key Laboratory of Materials–Oriented Chemical Engineering, College of Food Science and Light Industry, Nanjing University of Technology, Nanjing, China*

^2^ *State Key Laboratory of Bio-organic and Natural Products Chemistry, Shanghai Institute of Organic Chemistry, Chinese Academy of Sciences, Shanghai, China*

*Co-corresponding Author: **Hong Xu** and **Jiahai Zhou**

Supporting Information Legends ………………………………...S2-S4

Table S1………………………………………………….........…..S5

Figures S1-S9……………………………………………………..S6-S14

**Supporting Information Legends**

**Figure S1. The interactions between the N-domain (green) and the C-domain (yellow).** Hydrogen bonds are represented as red dashed lines while salt bridges are shown as blue lines.

**Figure S2. Sequence alignment of residues near the magnesium-binding site in NX-5, SmuA, PalI and MutB,** Secondary structure elements of NX-5 are shown above the alignment results. The residues involved in magnesium–binding of NX-5 are identical in other species and highlighted by green triangles.

**Figure S3. Structure-based sequence alignment of sucrose isomerases.** Sequences of SIases from *Erwinia rhapontici* NX-5 (NX-5, NCBI accession: ADJ56407.2), *Protaminobacter rubrum* (SmuA, ZP_16219852.1), *Klebsiella* sp. LX3 (PalI, AAK82938.1), and *Pseudomonas mesoacidophila* MX-45 (MutB, ABC33903.1) were aligned by ClustalW2. The secondary structure elements of NX-5 are depicted as arrows and helixes above the sequence. The substrate binding residues are indicated with blue triangles while the aromatic residues Phe297 and Phe321 are identified with black triangles. The catalytic residues Asp241 and Glu295, as well as the RXDRX motifs are highlighted by red triangles.

**Figure S4. Comparison of residues in the active site between native NX-5 (yellow) and native MutB (cyan).** The residues from NX-5 are labeled.

**Figure S5. View of the active site in the NX-5 mutant complexes.** (A) The binding mode of D-glucose in the active site of D241A. The electron density 2Fo-Fc maps are contoured at 1 σ and the water molecule was showed as a cyan ball. (B) Sucrose and D-glucose molecules binds to the active sites of E295Q/sucrose and D241A/ D-glucose.

**Figure S6. Structural alignments of native NX-5 and its saccharide complex.** (A) Structural comparison between D241A/glucose and native NX-5, residues from D241A are labeled. (B) Structural comparison between E295Q/glucose and native NX-5, residues from E295Q are labeled.

**Figure S7. Structural alignment of various SIases.** The alignment was shown in tube type of NX-5 (green), SmuA (cyan), PalI (yellow), MutB (magenta), and OGL (gray).

**Figure S8. Alignment of loop^330-339^ residues** **from SmuA (magenta), PalI (green), NX-5 (yellow), and MutB (cyan).**

**Figure S9. RMSD curves for 5 ns molecular dynamics (MD) simulations.** (A) RMSD curves for the complex of D241A/glucose and fructofuranose. (B) RMSD curves for the complex of D241A/glucose and fructopyranose. The RMSD changes of the complex, D-glucose, and fructofuranose/fructopyranose are represented by blue, black, and red lines, respectively.

**Table S1. Primers used for mutagenesis**.

| Mutation | Primers |
| --- | --- |
| D241A | F: 5’-GGTTTACGCTTTGCGACCGTTGCCACC-3’  R: 5’-GGTGGCAACGGTCGCAAAGCGTAAACC-3’ |
| E295Q | F: 5’-GCCACTGCGGGGCAGATATTTGGGGTT-3’  R: 5’-AACCCCAAATATCTGCCCCGCAGTGGC-3’ |
| F297A | F: 5’-GCGGGGGAAATAGCGGGGGTTCCTCTG-3’  R: 5’-CAGAGGAACCCCCGCTATTTCCCCCGC-3’ |
| F321A | F: 5’-ATAGCGTCTACGGCGGATCTGATCAGG-3’  F: 5’-CCTGATCAGATCCGCCGTAGACGCTAT-3’ |
| R325D | F: 5’-TTTGATCTGATCGATCTCGATCGTGAT-3’  R: 5’-ATCACGATCGAGATCGATCAGATCAAA-3’ |
| R328D | F: 5’-ATCAGGCTCGATGATGATGCTGATGAA-3’  R: 5’-TTCATCAGCATCATCATCGAGCCTGAT-3’ |
| ΔE332 | F: 5’-CGTGATGCTGATAGATGGCGGCGA-3’  R: 5’-TCGCCGCCATCTATCAGCATCACG-3’ |
| A330L | F: 5’-CTCGATCGTGATCTCGATGAAAGATGG-3’  R: 5’-CCATCTTTCATCGAGATCACGATCGAG-3’ |
| R335H | F: 5’-GATGAAAGATGGCACCGAAAAGACTGG-3’  R: 5’-CCAGTCTTTTCGGTGCCATCTTTCATC-3’ |
| R336T | F: 5’-GAAAGATGGCGGACCAAAGACTGGACC-3’  R: 5’-GGTCCAGTCTTTGGTCCGCCATCTTTC-3’ |
| K337I | F: 5’-AGATGGCGGCGAATTGACTGGACCCTT-3’  R: 5’-AAGGGTCCAGTCAATTCGCCGCCATCT-3’ |
| D338P | F: 5’-TGGCGGCGAAAACCTTGGACCCTTTCG-3’  R: 5’-CGAAAGGGTCCAAGGTTTTCGCCGCCA-3’ |
| W339R | F: 5’-CGGCGAAAAGACCGAACCCTTTCGCAG-3’  R: 5’-CTGCGAAAGGGTTCGGTCTTTTCGCCG-3’ |

**Figure S1**

**
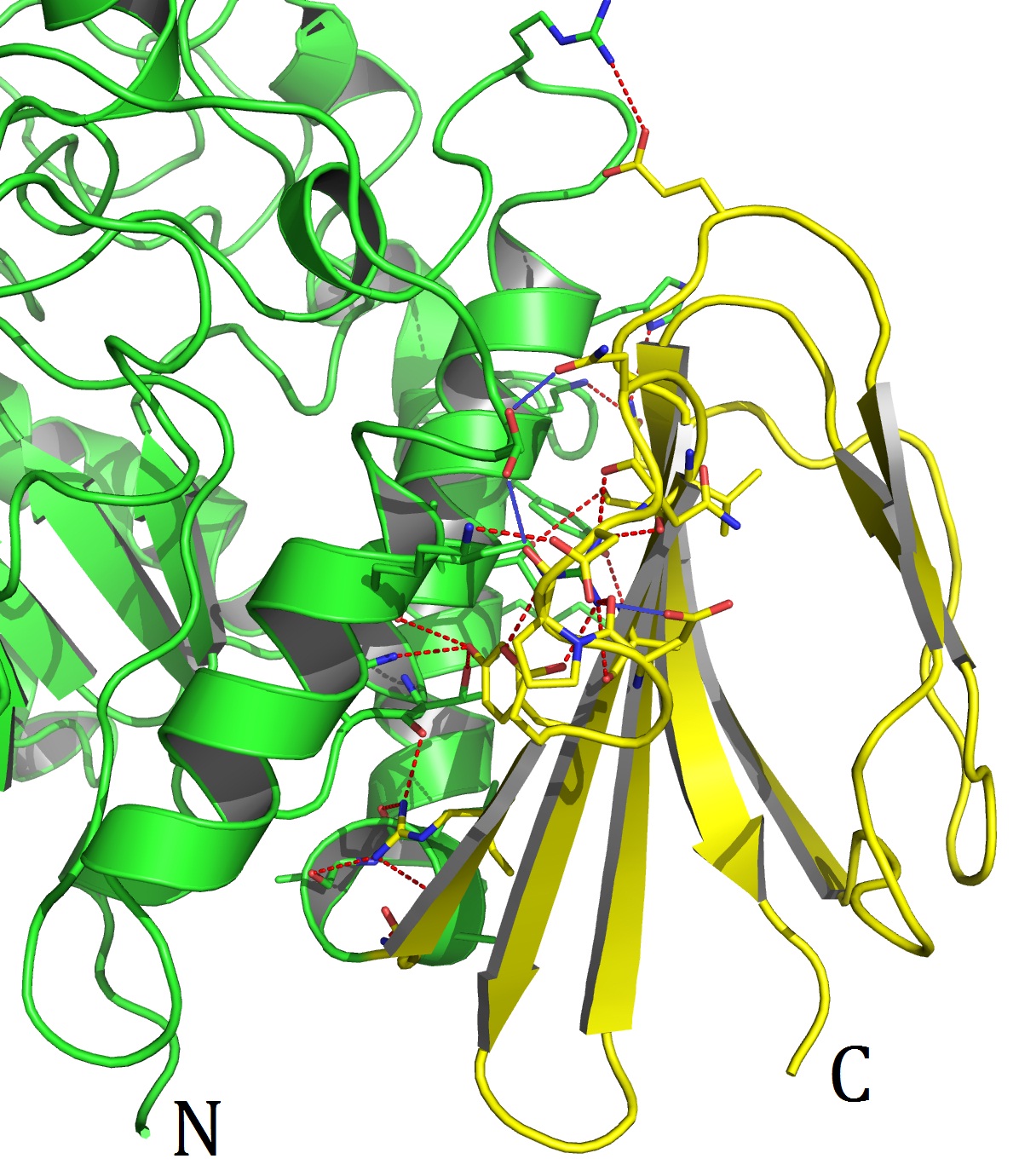
**

**Figure S2**


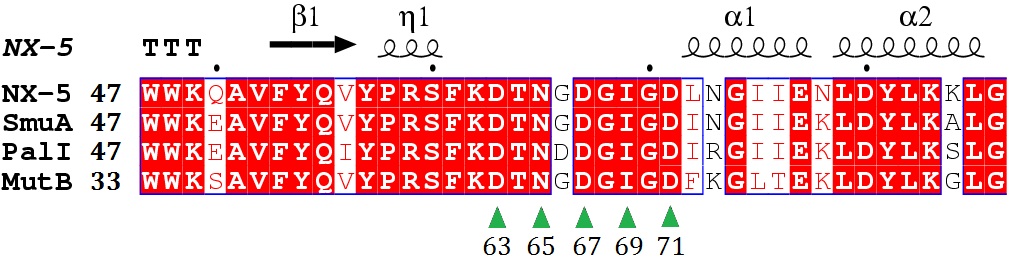


**Figure S3**


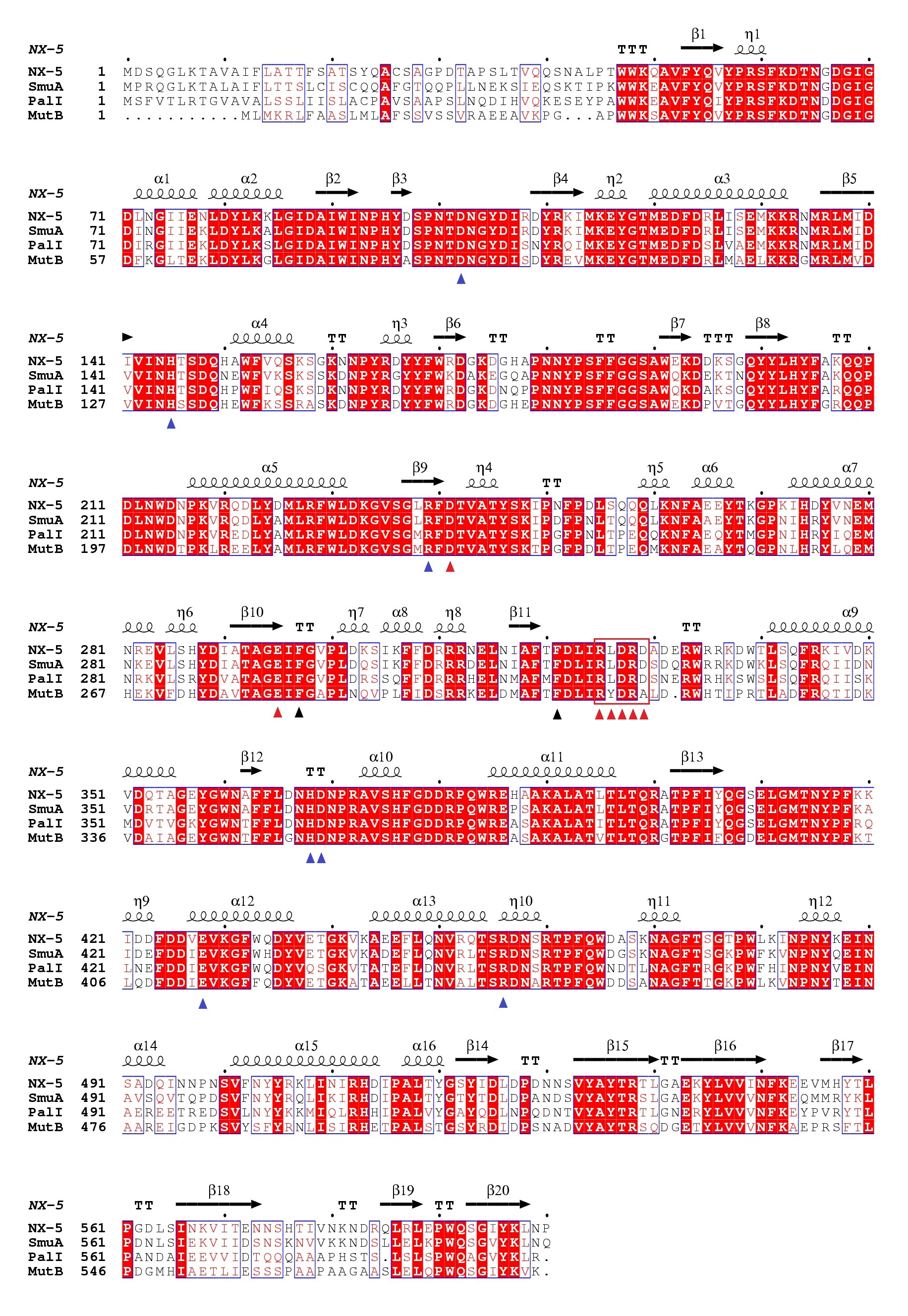


**Figure S4**


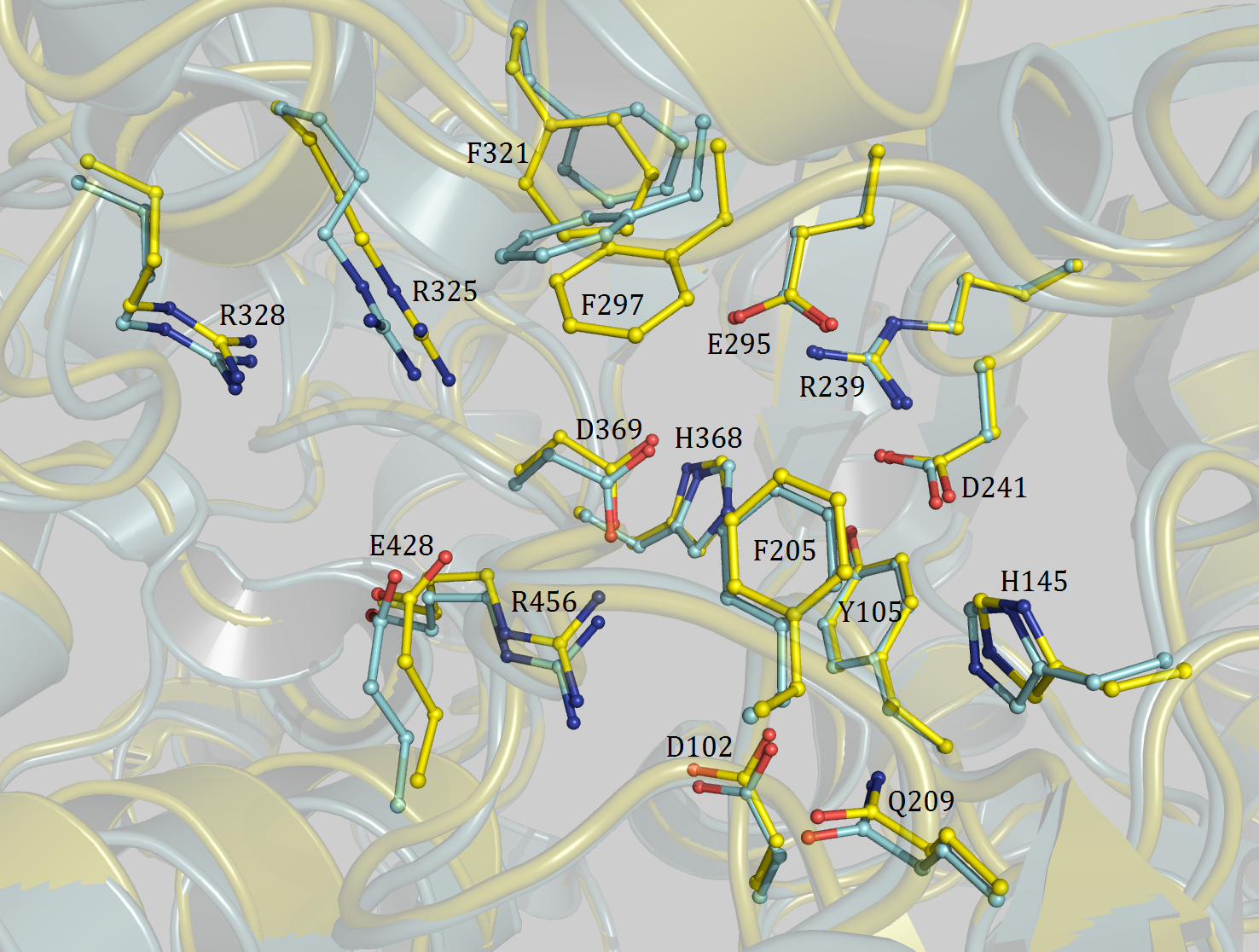


**Figure S5**

**
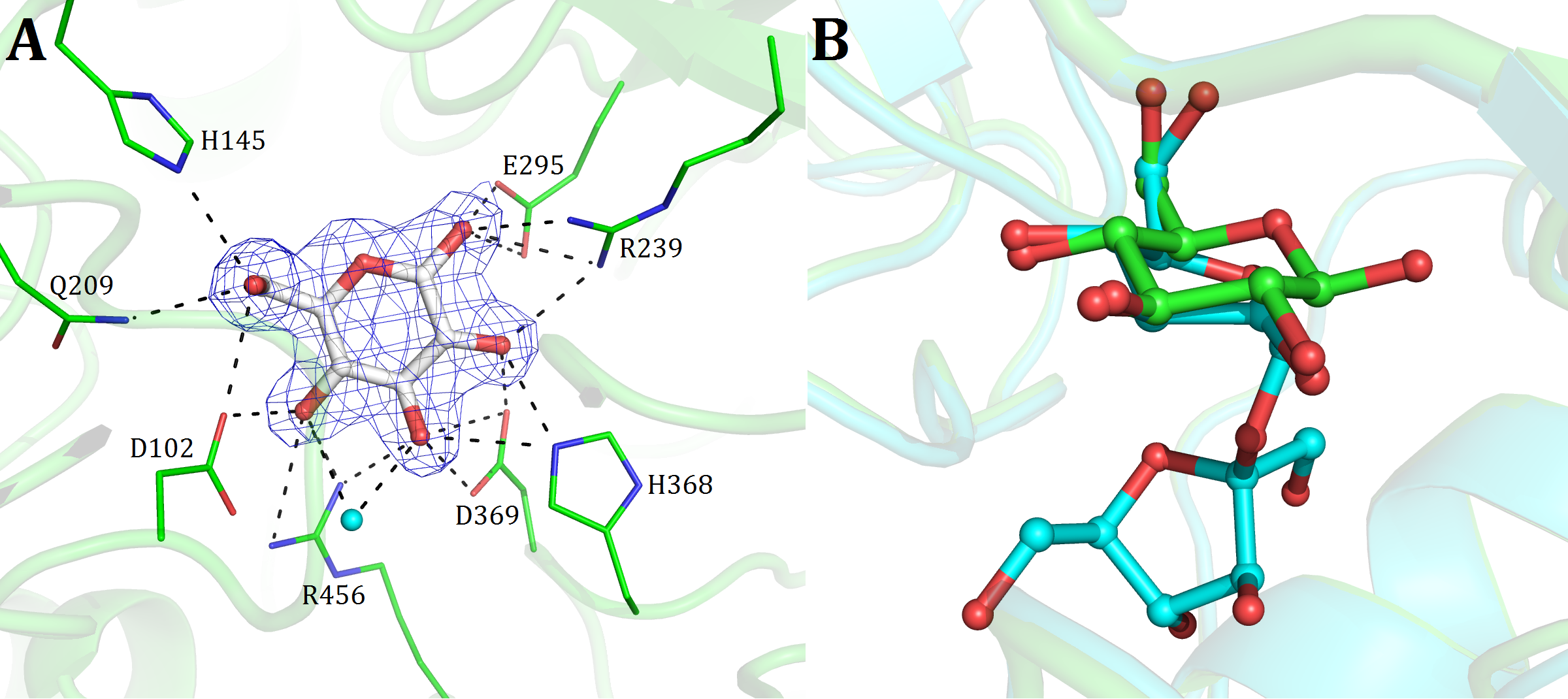
**

**Figure S6**

**
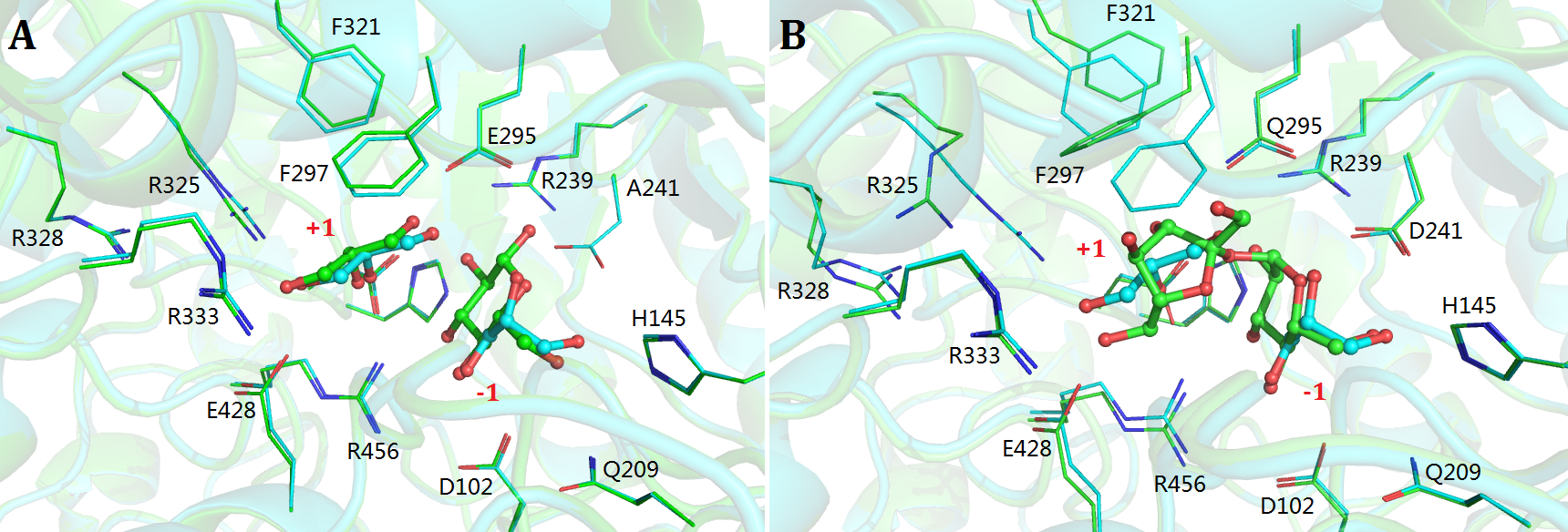
**

**Figure S7**

**
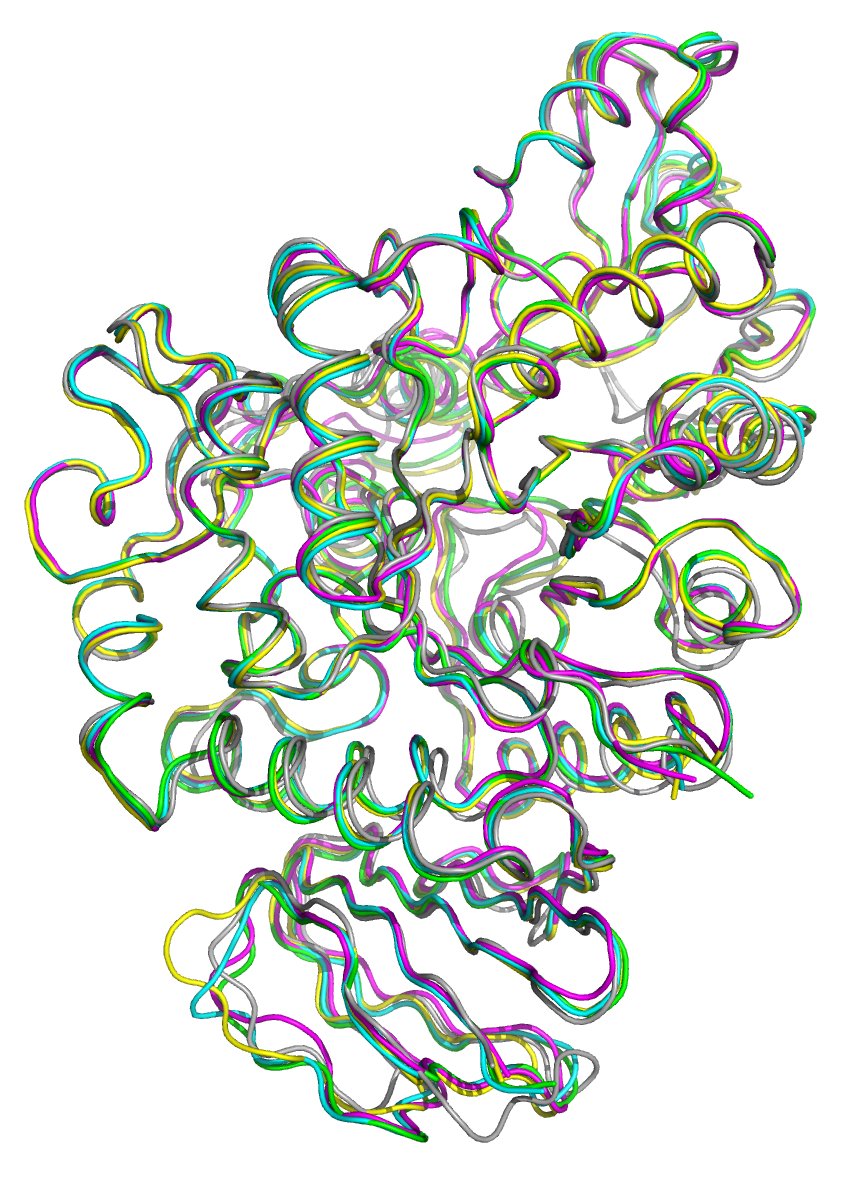
**

**Figure S8**

**
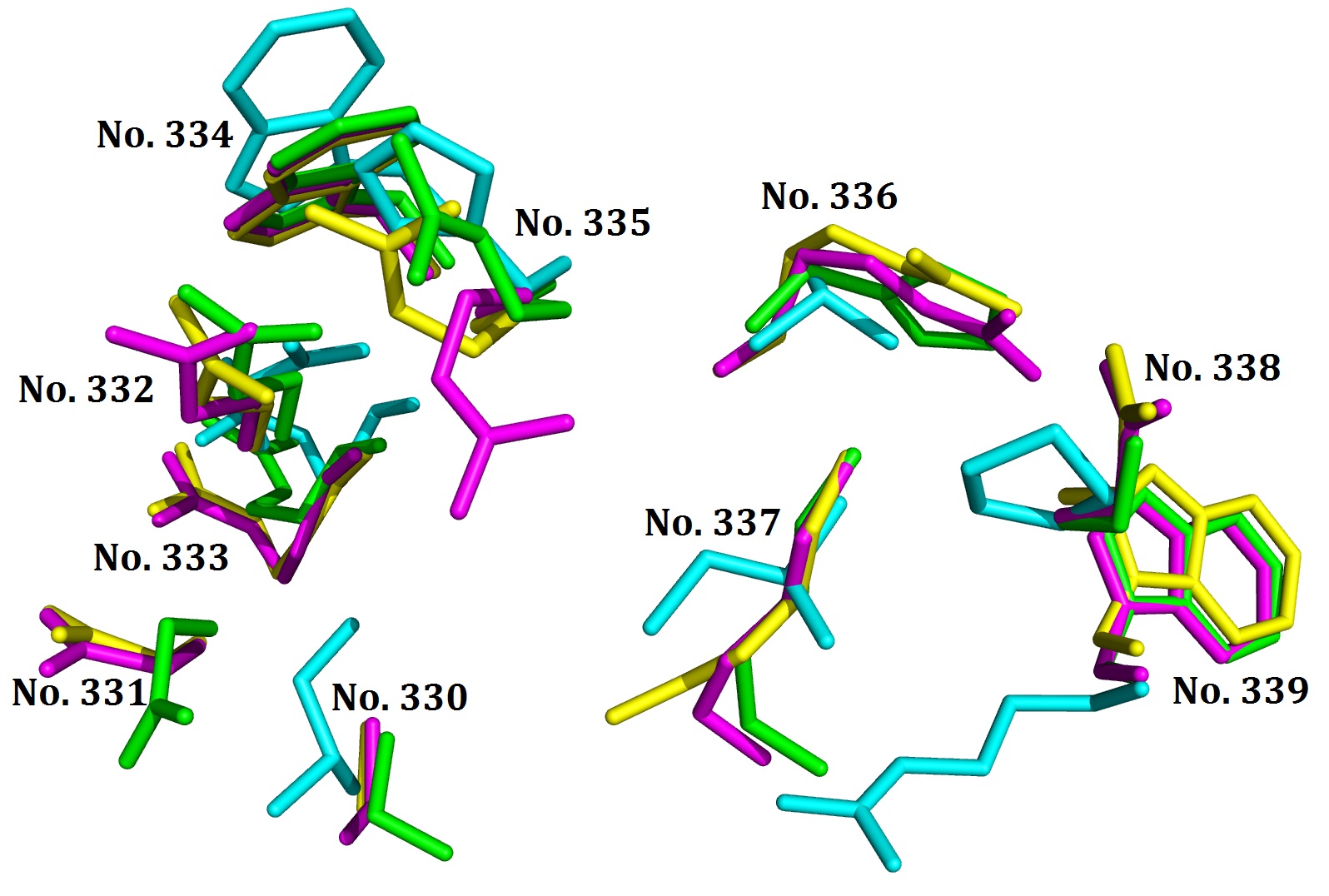
**

**Figure S9**

**
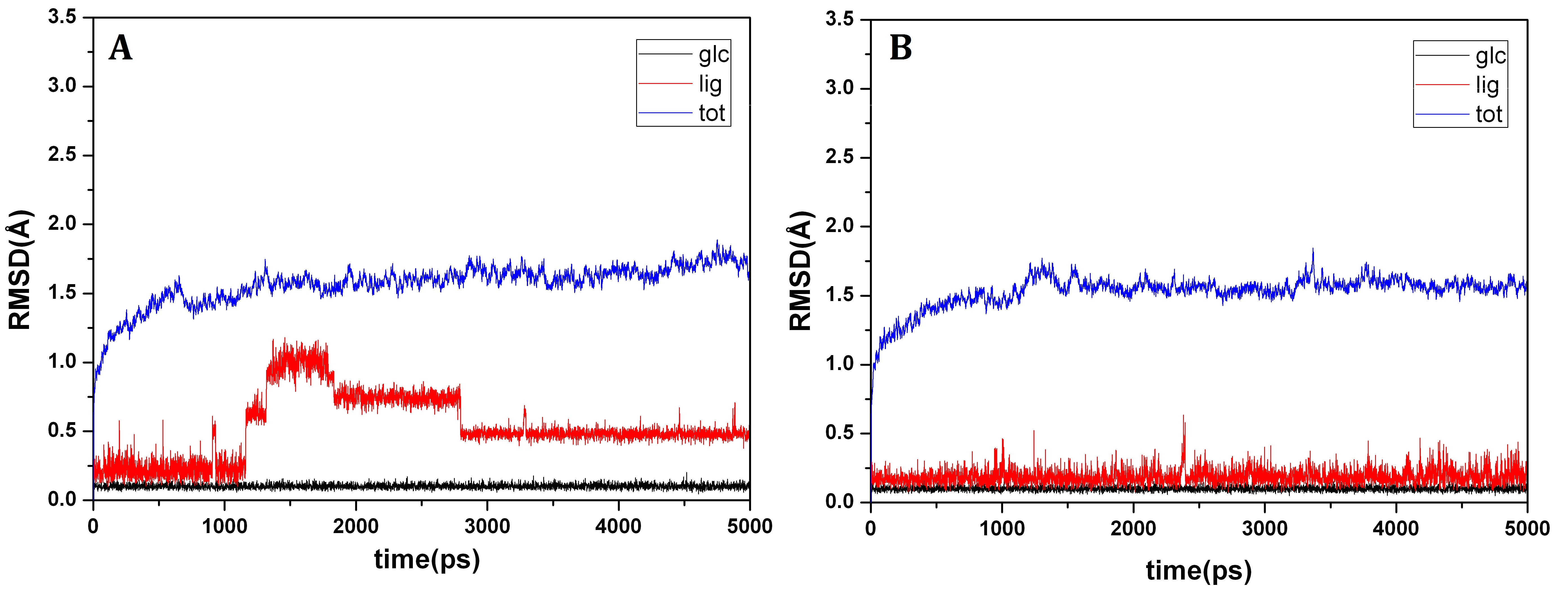
**
